# Development of the First Patient‐Reported Experience Measure (PREM) for Hearing Loss in Audiology Care—My Hearing PREM

**DOI:** 10.1111/hex.70088

**Published:** 2024-11-06

**Authors:** Sian K. Smith, Georgina Burns O'Connell, Rebecca Knibb, Rosemary Greenwood, Saira Hussain, Rachel Shaw, Jean Straus, Jonathan Banks, Amanda Hall, Nisha Dhanda, Sian Noble, Helen Pryce

**Affiliations:** ^1^ School of Optometry Department of Audiology, College of Health and Life Sciences Aston University Birmingham UK; ^2^ School of Psychology, College of Health and Life Sciences Aston University Birmingham UK; ^3^ Institute of Health & Neurodevelopment Aston University Birmingham UK; ^4^ Health Sciences University of York York UK; ^5^ University Hospitals Bristol and Weston NHS Foundation Trust Bristol UK; ^6^ Patient and Public Involvement Lead, HeLP research study; ^7^ National Institute for Health Research, Applied Research Collaboration West (NIHR ARC West) University Hospitals Bristol and Weston NHS Foundation Trust Bristol UK; ^8^ Population Health Sciences, Bristol Medical School University of Bristol Bristol UK; ^9^ Institute of Applied Health Research, College of Medical and Dental Sciences University of Birmingham Birmingham UK

**Keywords:** audiology, hearing loss, lifeworld‐led approach, PREM, qualitative research, stakeholder engagement

## Abstract

**Context:**

Patient‐reported experience measures (PREMs) provide important insights into the challenges experienced when living with a chronic condition. Although patient‐reported outcome measures (PROMs) exist in audiology, there are no validated PREMs to help clinicians understand patient perspectives and identify areas where patients may need additional support or interventions.

**Objective:**

The aim of this study was to develop and evaluate content for the new ‘My Hearing PREM’, which captures lived experiences of hearing loss from patients’ perspectives.

**Design:**

My Hearing PREM was developed and tested in two key phases. Phase 1 involved generating the PREM prototype in accordance with our conceptual model of the lived experience of hearing loss. In Phase 2, cognitive interviews were conducted with adults with hearing loss to appraise the content of the PREM (relevance, clarity, acceptability and comprehensiveness) and assess its respondent burden. Key stakeholders (e.g., adults with hearing loss, patient and public representatives, clinicians and researchers) were consulted throughout Phases 1 and 2 to review and refine the PREM. Interview data were analysed using thematic analysis.

**Setting and Participants:**

Sixteen participants (aged 16 years and over) with hearing loss took part in cognitive interviews, recruited from UK audiology departments and non‐clinical settings (e.g., lip‐reading classes, national charity links and social media).

**Results:**

Most PREM items were found to be relevant, clear, acceptable and comprehensive. Several problems were identified, including items not working well with the response scale options, irrelevant questions and a lack of clarity about terms (e.g., healthcare professionals) and whether questions should be answered based on the use of hearing aids (or not). The PREM was amended accordingly.

**Conclusions:**

Currently, no hearing loss‐specific PREMs exist in audiology. Involving multiple stakeholders in the development of the PREM helped to ensure that the items were relevant, clear, acceptable and comprehensive. The PREM is undergoing further evaluation and refinement in preparation for investigating the feasibility of implementing it into clinical practice.

**Patient or Public Contribution:**

Ongoing Patient and Public Involvement and Engagement (PPIE) with key groups (South Asian Women's groups, young people's groups, learning disability networks and student populations) was integral to the study. PPIE members reviewed patient information sheets and consent forms, advised on recruitment, reviewed the interview schedule and checked coding and analysis procedures. PPIE members provided feedback on the PREM's comprehensibility. Members of the public, including adults attending lip‐reading classes and hearing aid users from the South Asian community, provided feedback on iterative PREM drafts.

## 1 Introduction

Worldwide, more than 1.5 billion people (one in five) are affected by some form of hearing loss [1]. By 2050, this number could rise to 2.5 billion (one in four people), with nearly 700 million (one in 14) experiencing moderate or higher levels of hearing loss [1]. Hearing loss sharply increases with age and is the third leading cause of disability worldwide [2].

Living with hearing loss involves two types of work: coping with hearing loss in different social and listening situations (illness work), and seeking help and managing interventions (treatment work) [3, 4]. Illness work involves drawing on one's cognitive resources to make sense of hearing loss—interpreting symptoms, searching for the cause and considering how one's hearing might progress in the future [5, 6]. Socialising with others can be tiring and require additional cognitive effort (listening and concentration), which can result in withdrawal from or avoidance of social situations [3, 7]. The emotional burdens, including social disconnection, poor sense of identity and stigma, are well documented, yet may be unseen by others [3, 8, 9]. Inherent to the experience of hearing loss is coping with emotions such as anger, embarrassment and frustration [10]. Treatment work involves understanding options, learning how to use and adjusting to hearing technology, such as hearing aids [11, 12]. As we get older, co‐morbidities steadily accumulate [13], yet a person's capacity to manage health conditions (i.e., their affective, cognitive, material and social resources) may diminish [14].

Important strides have been made in audiological care to promote patient choice and autonomy [15, 16], alongside more widespread developments in patient‐centred care [17, 18, 19]. Patient‐centred care empowers individuals to actively participate in all aspects of their hearing care, with healthcare professionals and significant others providing support to help them make informed decisions that align with their values [16, 20]. Key components of this approach include demonstrating empathy, active listening, asking open‐ended questions, understanding patients' needs and preferences and fostering shared decision‐making [21, 22]. Importantly, evidence shows that shared decision‐making benefits patients and healthcare services more generally in terms of saving time and aiding disadvantaged groups [21, 23, 24].

To complement and enhance the delivery of patient‐centred care, healthcare professionals can explore the person's lifeworld [25, 26]. This means understanding their subjective (emotions, opinions and beliefs) and social world (relationships and cultural rules), alongside objective world (medical evidence related to treatment options) [25, 27, 28]. In practice, this means asking different questions in healthcare encounters, which relate to lived experience, rather than focusing solely on health outcomes [25]. Lifeworld‐led care adopts a holistic approach, both in the type of evidence‐guiding practice—primarily qualitative research focused on lived experience—and in the way care is delivered. Enhancing patient‐centred care with this lifeworld‐led approach positions individuals as agentic citizens who are empowered to make care and treatment decisions within the context of their wider lives, not just to achieve the best health outcomes [27, 29].

The current study forms part of a larger research programme—the Hearing Loss and Patient Reported Experience (HeLP)—which seeks to develop and validate a pragmatic tool for use in audiology care and research: a patient‐reported experience measure (PREM) designed to capture the lived experience of hearing loss and the hidden work required to manage it [30, 31]. Our vision for the PREM is to facilitate lifeworld‐led dialogue between patients and staff, focusing on the patient experience of hearing loss so that we can provide a mechanism through which patient‐centred and lifeworld‐led approaches can be integrated into audiology care.

Self‐report questionnaires are increasingly used to capture patient perceptions of care and treatment to use them to improve the quality of care. Commonly used questionnaires include patient satisfaction measures, patient‐reported outcome measures (PROMs) and PREMs [32]. Existing patient satisfaction measures assess whether patients’ evaluations of care match their expectations, for example, ‘Did the audiologist give you sufficient information about your hearing loss?’ In contrast, PROMs are designed to elicit patient views on their symptoms and treatment [33]. In audiology, PROMs are commonly used to measure functional changes (e.g., access to additional sounds via hearing aids) and how satisfied patients feel about their hearing technology [34, 35]. They are also used to measure the perceived amount of effort required to listen [36] and evaluate the functional impact of hearing loss on social participation (e.g., group discussion) [37]. More recent work has focused on patient empowerment in hearing healthcare [38, 39]. In contrast, PREMs focus on patient experience rather than health outcomes. They capture the experience of living with a health condition and receiving care [40, 41, 42]. Although PROMs are developed and routinely used in audiology [35], there qare currently no validated PREMs. PREMs can enhance communication between patients and audiology staff by allowing patients to express their experiences and concerns. This information can help to identify areas where patients may need additional support, informing changes in practice [43, 44].

This article presents two key phases of the broader HeLP study [1]: developing a PREM prototype based on our published conceptual model of the lived experience of hearing loss [45], and [2] testing the PREM prototype with adults with hearing loss using ‘think aloud’ cognitive interview methods to evaluate the content of the PREM (relevance, clarity, acceptability and comprehensiveness) and its respondent burden. Throughout Phases 1 and 2, key stakeholders—including adults with hearing loss, patient and public representatives, clinicians, researchers and health commissioners—were involved in reviewing and refining the PREM, known as ‘My Hearing PREM.’ This work was produced by the Hearing Loss and Patient Reported Experience study team/Aston University and University Hospitals Bristol and Weston NHS Foundation Trust.

## 2 Methods

### 2.1 Design

**Figure 1:**
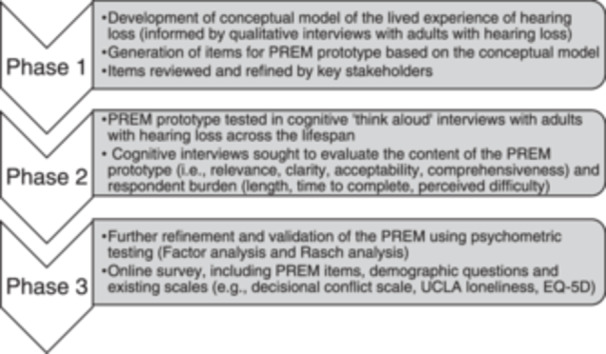
Overview of PREM development and testing process. Phases 1 and 2 are the focus of the current article, whereas Phase 3 will be reported in a separate publication.

In the absence of PREM guidance, we relied on existing audiology PROM development literature to inform our PREM development [46, 47, 48]. In Phase 1, we developed a clear conceptual framework of the lived experience of hearing loss, which is detailed in a separate publication (Figure 1) [45]. Using this conceptual model, we generated items for the PREM prototype. In Phase 2, key stakeholders reviewed and refined the prototype, followed by cognitive interviews with adults experiencing hearing loss. These interviews evaluated the questionnaire's content (relevance, clarity, acceptability and comprehensiveness) and respondent burden, including ease of understanding, length and time required for completion [49]. Although not the focus of the current article, the next step (Phase 3) is to further refine and evaluate My Hearing PREM using psychometric testing [50], which will be published separately.

This study was approved by the West of Scotland Research Ethics Service (approval date: 6 May 2022; ref22/WS/0057) and the Health Research Authority and Health and Care Research Wales (HCRW) (approval date: 14 June 2022; IRAS project ID: 308816).

### 2.2 Stakeholder Engagement

During Phase 1 (Development of the PREM prototype) and Phase 2 (Cognitive testing), key stakeholder groups (detailed later) were invited, either online or in person, to provide feedback on the acceptability and clarity of the evolving PREM. Clinicians and researchers were consulted about the feasibility of using the PREM for clinical and research purposes, respectively. Clinicians were also asked whether it captured important aspects of care and whether any of the concepts might cause distress for patients.

i. Adults with hearing loss, including adults with lived experience who were part of our Patient and Public Involvement and Engagement (PPIE) groups (described later), and members of the study's steering committee group.
ii. Clinicians (working in public health, hearing therapy and audiology) and a Clinical Commissioning Group (CCG) representative.
iii. Researchers (from psychology, sociology, health economics, medical statistics, audiology and health science disciplines).

### 2.3 PPIE

PPIE was a critical component in developing My Hearing PREM. Our two PPIE leads (a researcher with PPIE responsibility and an expert by experience) organised and managed PPIE activities, which included reviewing patient information sheets, advising on recruitment and strategy, reviewing the PREM items and interview schedule and checking coding and analysis procedures. We invited and sought feedback from under‐represented groups (South Asian Women's groups, young people's groups and learning disability networks) as well as volunteers from hearing loss networks, hearing therapy students, attendees at lip‐reading classes and health commissioners to provide feedback on the PREM's format, content, and comprehensibility throughout Phases 1 and 2 (Table 1). Individuals from our PPIE groups were contacted in person, online, via phone and through online meetings. Items were revised, added or removed iteratively in response to PPIE feedback.

**Table 1:** Questions asked to obtain feedback on the PREM items from PPIE members and stakeholders.

Are these questions meaningful to your experience of hearing loss?
Are these questions different to those you have been asked in your clinical experience?
What are we missing?
What is most important for us to ask?
How easy are the questions to read?
Do you understand what we are trying to find out?
Would you fill this out?

### 2.4 Phase 1: Development of the PREM Prototype

PREM items were generated using our evidence‐based conceptual model, developed through a synthesis of the literature (article forthcoming [51]) and qualitative interviews with adults (aged 16 years and over) living with hearing loss recruited from different age bands (16–29, 30–49, 50–79 and 80 upwards) [45, 52]. Participants were eligible if they could provide fully informed consent and communicated using spoken language. They were invited regardless of whether they used hearing aids or not.

Central to our model was the core category, *individualised responsibility*, which represents the personal responsibility experienced by individuals when coping with hearing loss [45]. *Individualised responsibility* helps to explain variation in the types of experiences reported by individual and is dependent on the following four surrounding components that manifest in the lived experience of hearing loss:

i. *Individualised auditory lifeworld:* Being responsible for what and how you hear.
ii. *Individualised patient‐centred care*: Being responsible for seeking help and enacting interventions.
iii. *Social comparison and social support*: Being responsible for recognising differences and seeking out support.
iv. *Individual agency and capability*: Being responsible for making the efforts (cognitive and emotional) to cope with hearing loss.

These components influence how individual responsibility for hearing loss is experienced, and the degree to which burden and distress are experienced (e.g., high levels of social support that ‘normalise’ the hearing loss experience reduce the distress associated with individual responsibility). Conversely, low social support and lower individual capability may increase the distress associated with the individualised responsibility of hearing loss and expectations of self‐management.

Members of the research team (S.S., G.B.O.C., H.P. and S.H.) independently developed a list of potential PREM items based on our conceptual model and interview data. The team met regularly online to present and discuss these items. Any discrepancies regarding item inclusion or wording were resolved through ongoing discussions focused on the item's relevance, clarity and alignment with the conceptual model. The goal was to create concise items that were understandable and interpreted as intended. Where possible, participants’ language was used to phrase the items. The readability of the items was assessed using Flesch–Kincaid Reading Ease [53] and Flesch–Kincaid Grade Level formulas [54]. Higher scores (on a scale of 0–100) indicate easier reading. The generated score corresponds to the US grade level required for text comprehension. For public texts, the target should be a grade level around 8, equivalent to year 9 in the UK (ages 13–14 years) [55].

Given the focus on the lived experience of hearing loss, the PREM did not include items about hearing aids. We wanted to ensure the PREM could be completed by anyone with hearing loss, regardless of their hearing aid use. This approach acknowledges that although hearing aids influence the experience of hearing loss, they do not eliminate all hearing or communication difficulties [45]. Furthermore, there are existing PROMs that assess the benefits of hearing aids, making it unnecessary to duplicate efforts within the PREM [36, 56].

### 2.5 Phase 2: Cognitive Testing of the PREM Prototype

#### 2.5.1 Participants

We invited participants from our initial qualitative interviews (conducted as part of the HeLP study to develop our conceptual model) to take part in the cognitive interviews. A diverse group of male and female participants from different age ranges were included. These participants were originally recruited from clinical sites in England (Bath, Bristol) and Scotland (Tayside), as well as non‐clinical settings such as social media, lip‐reading classes, the university and residential care homes. Eligibility criteria included being 16 years or older, living with hearing loss, having the capacity to provide fully informed consent and communicating primarily through spoken language.

#### 2.5.2 Data Collection and Procedure

Cognitive interviews using ‘think aloud’ and ‘verbal probing’ methods were conducted to encourage participants to verbalise their thoughts about the PREM prototype [57, 58]. The content was appraised against the following criteria: *relevance* (representative of the lived experience of hearing loss), *clarity* (easy to understand and interpreted as intended), *acceptability* (deemed appropriate and sensitive to individuals with hearing loss) and *comprehensiveness* (captures important aspects of living with hearing loss) [38, 39, 47, 59].

An interview guide (Supporting Information S1: Material 1) sought to (i) identify participants’ understanding and interpretations of item wording and scoring; (ii) ensure items were relevant and reflected their experiences; (iii) identify participants’ ability to recall information to answer items and (iv) identify redundant, repetitive or missing items. We also asked participants to comment on ease of completion, general structure and length and suggestions for improvement. During this process, interviewers took notes to capture responses.

Interviews were carried out between June and September 2023 by four experienced qualitative researchers (H.P., S.H., G.B.O.C. and S.S.): two academic‐clinician researchers (a clinical scientist and a hearing therapist) and two research associates with a sociology background and health psychology background. All participants gave written informed consent. Interviews were conducted online using Microsoft (MS) Teams or in the participant's home, the university or the audiology clinic. Online interviews were transcribed using the MS Teams transcription feature and edited for accuracy. In‐person interviews were transcribed verbatim by a professional transcription service. All recordings were deleted once transcriptions were anonymised and edited for accuracy.

The interviews were conducted in two phases: 10 participants took part initially, and a different set of six participants took part later. Feedback from initial interviews (*n* = 10) was used to remove or reword items before the second set of participants (*n* = 6) were interviewed. Following scale development work, an item was considered for revision if two or more participants encountered similar issues with its relevance, acceptability, clarity or any other feature of the question [47].

#### 2.5.3 Analysis

The cognitive interview data were analysed using thematic analysis, as typical in previous scale development work [38, 39]. Deductive thematic analysis was used in accordance with the criteria against which the PREM items were reviewed (i.e., relevance, clarity, acceptability and comprehensiveness) [60]. Further inductive coding identified anything else not captured by those criteria [48]. Team meetings were held to check the analysis and discuss interpretations. We wanted to understand items that triggered emotional responses, how participants reflected on prior experiences related to an item, and responses to items on clinical care versus items on living with hearing loss. We also noted instances where participants sought clarification, whether the language used affected their responses, or if any responses seemed hampered by ambiguity or inconsistency in participants’ interpretations.

## 3 Results

### 3.1 Phase 1: Development of PREM Prototype

#### 3.1.1 Initial Item Generation

**Figure 2:**
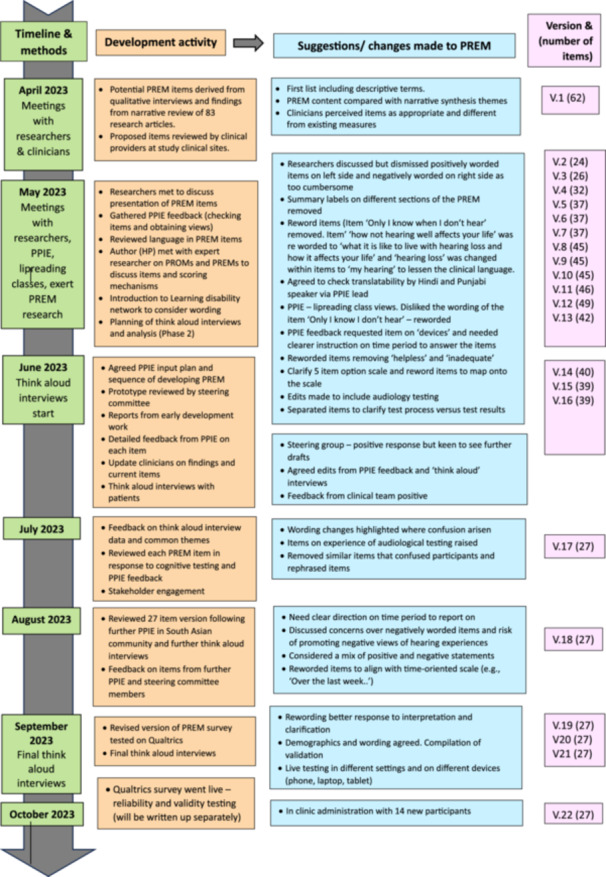
Timeline of PREM development activities across Phases 1 and 2.

Figure 2 presents a timeline of PREM development activities. The research team generated an initial pool of 62 items categorised in accordance with our conceptual model, developed through our literature review and in‐depth interviews with 46 adults with hearing loss aged between 16 and 96 years, of which 29 were female, and 34 wore hearing aids [45, 52]. Table 2 illustrates potential PREM items. We decided on a five‐point Likert‐type scale (Always, Most of the Time, Sometimes, Rarely and Never), enabling responses to be sensitive to variations in experience [61].

**Table 2:** Potential PREM items informed by the conceptual model of lived experience of hearing loss.

Conceptual category	Description of category	Quotes (supporting evidence from qualitative interviews)	Potential PREM items (examples)
Individual responsibility (core category)	The sense of individual responsibility for the management of hearing loss that falls to the patient.	*It's down to you to learn to deal with things and it's your problem (hearing loss), not anybody else's. (participant 43, age range 30*–*49 years)*	I feel the burden of my hearing loss is all on me
		*The onus is on that one individual in a social situation, where I don't know the other people, it is absolutely on me. (participant 17, age range 30*–*49 years)*	I feel I have to manage my hearing by myself
Individualised auditory lifeworld	The level of isolation experienced.	*If you don't hear everything, you tend to cut off. You go in your own world. (participant 34, age range 50*–*79)*	I feel so on my own when I can't hear and others can
		*It's isolating. It's a community (hearing world), I am sort of left out, a bit removed from things. I can't hear what he's (young son) hearing or I can't really talk to him. (participant 21, age range 30*–*49 years)*	There are certain situations where I feel really left out
		*You can feel a bit cut off, because some times I've missed a particular punchline or whatever, which I find really annoying. Someone's telling a joke and the punchline's right at the end and I miss it, sometimes they don't make it very clear or rush it, but I know I have to ask some. (participant 24, age range 80+ years)*	
Individualised auditory lifeworld	The sense of concern or fear for the future of their hearing loss.	*It's like, is it going to get any worse, because I asked the audiologist, obviously, because the left one's gone worse than the right now. (participant 45, age range 30*–*49 years)*	I worry my hearing will get worse in the future
		*I do think about it (how hearing might progress in the future) and I do worry about it. I have asked that audiology appointments about it and they never can tell me anything. I always feel like I'm having to say, you can see that I'm younger than your average person, so that's why I'm interested in this question and they don't really necessarily seem to think it's obvious. (participant 47, age range 30*–*49 years)*	A healthcare professional has talked to me about how my hearing might progress in the future
Social comparison and support	The level of social support with hearing loss (broader societal, but also family and friends type support).	*My friends are really good. I go out with them regularly and two friends, in particular, they've learnt the best way to sit which is if they both sit opposite me and I sit so I can see them to lip read. (participant 30, age range 50*–*79 years)*	I feel lonely when I can't hear and others can
		*My wife and my daughters and the grandchildren, they'll say something at the wall and I just hear something and I say, ‘I can't hear what you're saying’, so what they do is raise their voice, and I said, ‘Look, turn around, then I have got a chance of understanding what you're saying, lipreading and things.’ (participant 36, age range 50–79 years)*	There are certain situations where I feel really left out
Social comparison and social support	The sense of belonging individuals have to social groups (in hearing and deaf communities)	*I miss out on the chit‐chatter, whether it's at a restaurant, whether it's walking down a street with a group of friends, whether it's team away days (at work), you miss out on the general chit‐chat. (participant 10, age range 50*–*79 years)*	My family and friends support me with my hearing
		*It's kind of like a weird niche, isn't it? It's like partially deaf people don't quite fit*. *If* someo*ne was signing hello or whatever to me, I'm just like, “I'm sorry, I don't know that language.” I feel a bit of an alien to that, as an outsider, I fear that I wouldn't feel welcome. I feel that I would be denigrating that effort to like get together as a deaf men's group. (participant 46, age range 30‐49 years)*	Medical/healthcare professionals support me with my hearing
		*l*	
Individualised patient‐centred care	Negotiating healthcare encounters (seeking help, interaction with healthcare professionals)	*So, one of the audiologists was also a lip‐reading teacher and had lived experience, had hearing loss herself. And if I call with questions or problems, I can go to them and they are accessible.participant 17, age range 30*–*49 years)*	I feel confident communicating my concerns and needs to healthcare professionals
		*iNobody sits down and say it's OK. You know this is going to be really difficult and ther*e *was not health professional I had access to that I could talk to about hearing loss. (participant 31, age range 50‐79 years)*	I am confident that healthcare professionals will listen to my point of view
Individual agency and capability	The level of information and knowledge they have to assist with decisions.	*So, by this time, I have been struggling with a major hearing loss for eight months. I don't know what to ask for. I don't know what's available. I have no idea and you trying to help me when you don't have any idea and I don't have any idea and I feel stupid because I don't know if this is temporary or permanent. My husband has stopped talking to me. It's a waste of time to talk to you. You can't hear. You know, I'm feeling phenomenally isolated and it must be my fault because it's my hearing that has gone. Can't possibly be anybody else's fault. (participant 31, 50*–*79 years)*	I know the different things that can help me with my hearing like devices and support
			It would be helpful to be directed to services that can help my hearing such as lip‐reading classes, groups or hearing therapy
Individual agency and capability	The sense of frustration in communication.	*And they don't understand you feel frustration, you know, they think, ‘why isn't he listening to me? Or I told him that just now’. You know, (participant 23, age group 80+ years)*	I am frustrated by my hearing problems
		*I find it really difficult and frustrating and upsetting, I guess when people like, if you do mishear something or you do, you don't hear something and you ask someone to say like say it again and they say ‘ohh, don't worry’, you know or kind of dismiss it. (participant 12, age range 16–29 years)*	I find it hard to communicate with services because of the difficulty hearing them (e.g., companies, organisations, GP surgeries, etc.)
Individual agency and capability	Doing the work of hearing loss (cognitive, emotional and physical efforts)	*I had a training course for work. I was knackered afterwards. I do get very tired (with my hearing). My brain is completely fried, my ears and my eyes just don't want to know at all. So, I just usually sit and watch telly to unwind and calm down. But, it takes me a few days to really recover. (participant 13, age range 50–79 years)*	Trying to hear can be exhausting
		*I would say having a hearing impairment, because you get tired with your concentration, it's a bit like speaking a second language. So, the challenges of processing and you know hearing and interpreting processing, they're similar to somebody with a second language. (participant 11, age range 30–49 years)*	I have to concentrate more because of struggling to hear

#### 3.1.2 Stakeholder Review

Forty‐five stakeholders, including 21 adults with hearing loss, 18 clinicians and six researchers, reviewed My Hearing PREM. Stakeholders were consulted because of their expertise; they were not research participants. In this role, stakeholders discussed how the new PREM differed from current PROMs. Clinicians were particularly interested in adding personalised data on individual experiences to clinical encounters. They considered that the items were unique and addressed important aspects not typically included in standard audiology assessments.

Discussions included whether items should reference specific situations, the impact of positively and negatively worded items and the potential to enhance patients’ decision‐making. Health service commissioners raised concerns that negatively worded items might undermine confidence in self‐management, whereas clinicians felt that they allowed individuals to express negative experiences. To address concerns, some items were edited to reduce negative language. All stakeholders agreed that the PREM was clear, well‐written and valuable for tailoring care and facilitating important conversations.

#### 3.1.3 PPIE Review

Fifty‐six individuals from our PPIE groups reviewed My Hearing PREM. As mentioned earlier, PPIE members are not research participants, which means we cannot report their characteristics beyond the groups they represent.

Overall, the concepts underpinning the PREM items resonated with their experiences. They particularly appreciated a greater emphasis on residual difficulties, such as having to remind others how to communicate (e.g., getting their attention before talking to them and speaking face to face) rather than questions focused on functional change. PPIE respondents from the South Asian community inquired about the translatability of the PREM to Punjabi, Hindi and Urdu languages. Broadly, the PREM was found to be translatable but there were specific suggestions for item changes. The phrase ‘making sense’ was suggested for removal in favour of ‘understand’. There were some concerns about the five‐point scale and that ‘some of the time’ could be difficult to translate, so it was reworded to ‘sometimes’. After discussion, it was decided to retain the five‐point scale for the reliability and validity testing, and this would be unlikely to affect overall scoring and discrimination of people who were experiencing more and fewer negative experiences.

PPIE respondents identified gaps in earlier drafts of the PREM. Specifically, the challenges of being out in public and feeling safe. This concern was also raised in our interview data; therefore, an item was added to capture feeling unsafe when out and about. Our PPIE lead and co‐author reported feeling apprehensive about audiology appointments and getting her hearing tested [62]. Likewise, interview data indicated anxiety about testing, and items were added to reflect this concept.

Another PPIE respondent described a scenario in which their hearing loss became a significant problem when their car broke down and she needed to call a rescue service only to find a pre‐recorded message. In response, we added an item: ‘I find it hard to communicate with services because of the difficulty hearing them e.g., organisations, GP surgeries, etc.’

During PPIE feedback, several items were revised to enhance their clarity (e.g., ‘I often talk about the effect of my hearing loss with other people’ and ‘I feel cut off from family and friends at social gatherings’). These revised items were taken back to the PPIE for further consultation; where more than one interpretation of an item was identified, the item was reworded accordingly.

Some wording was value laden, such as ‘must’ in the item ‘I must try hard to hear’. This item was deemed unclear and potentially harmful by reinforcing negative thoughts. Similarly, PPIE respondents advised against using the term ‘hearing loss’ too frequently because of the stigma embedded within this deficit model [63]. We revised items using the term ‘hearing’ rather than ‘hearing loss’. We used the term ‘problems’ to clarify the item ‘I am frustrated by my hearing problems’, but otherwise worded items to use ‘hearing’ as a non‐deficit loaded descriptor.

Before cognitive testing, we iteratively reworded or removed items that were open to multiple interpretations, or used words considered pejorative, negative or difficult to understand. We rephrased some of the negatively worded items (e.g., ‘I feel like others do not understand my hearing’) to positively worded items (e.g., ‘I feel like others understand my hearing’). This meant the questionnaire included reverse‐worded items (items phrased in the opposite direction to other items).

Overall, a total of 40 items remained after the stakeholder and PPIE review and were subsequently tested in cognitive interviews. Table 3 presents the first 15 PREM items before and after the cognitive interviews.

**Table 3:** Example PREM items—the first 15 items before and after the cognitive interviews (versions 14 and 22, respectively).

Example PREM items (V.14)—pre‐cognitive interviews	Example PREM items (V.22)—post‐cognitive interviews
1. I feel confident to say if I haven't heard someone/something	1. I feel confident telling people when I haven't heard them
2. Other people don't seem to realise when I have not heard something	2. It is an ongoing struggle to hear others
3. I have to work hard to communicate with others (e.g. ask people to repeat themselves)	3. I am frustrated by my hearing problems
4. I am frustrated by my hearing problems	4. I feel lonely when I can't hear and others can
5. I am confident in social situations with my hearing	5. When I speak to people for the first time, I tell them about my hearing
6. My hearing makes me feel lonely	6. There are certain situations where I feel really left out
7. I talk to others about how my hearing affects me	7. I avoid activities I used to enjoy because of my hearing
8. I feel left out from other people at social gatherings or work	8. I have to concentrate more because of struggling to hear
9. Family and friends are helpful with my hearing	9. I worry what people think of me because I can't hear everything
10. I avoid activities I used to enjoy because of my hearing	10. I am confident talking to people in background noise
11. I have to concentrate more because of my hearing	11. I notice the problems caused by not hearing
12. I feel like others do not understand my difficulties with hearing	12. Over the last week, I have worried my hearing will get worse in the future
13. I worry about what others think of me because of my hearing (e.g., family and friends or people I meet)	13. Over the last week, I have felt unsafe when I am out because I don't hear what is going on around me
14. I understand why I hear the way I do	14. Trying to hear can be exhausting
15. I can access practical solutions to help me (e.g. digital solutions, captions or devices)	15. Family and friends support me with my hearing

### 3.2 Phase 2: Cognitive Testing of PREM Prototype

#### 3.2.1 Sample Characteristics

A total of 16 participants with representation across the life course took part in the ‘think aloud’ cognitive interviews (Table 4). Ten were female, 15 were hearing aid users, and 4 were also clinicians working in audiology.

**Table 4:** Sample characteristics (*N* = 16).

Characteristic	*n*
Age group	
16–29 years	3
30–49 years	3
50–79 years	4
80 years—end of life	4
Gender	
Female	10
Male	6
Hearing aid user	
Yes	15
No	1
Seeking clinical help for hearing	15
Not currently seeking clinical help	1

#### 3.2.2 Themes

Deductive analysis of data across the four themes (relevance, clarity, acceptability and comprehensiveness) is reported below and in Table 5. A fifth theme regarding the conceptual overlap of items was also identified.

**Table 5:** Examples of items revised or removed during the think‐aloud interviews in accordance with the themes.

Theme	Sample items	Findings and issues	Quote	Outcome for item
Relevance	I feel detached from the world at times because I miss out on news broadcasts and public announcements	Participants did not relate to the term ‘detached’	*I don't feel unnecessarily miss out on that. I'm kind of a generation where technology and like smart tech has been available to us. (TA6, female, 30*–*49 years)*	Removed
		Felt the item was more relevant for older generations as younger generations mostly use technology/social media to access news	*In today's social media, it comes to you from all angles, doesn't it? (TA8, female, 80+ years)*	
			*I get like all my news broadcasts off my phone though with my eyeballs. I have little feeling for that question. (TA9, male, 30*–*49 years)*	
Relevance	My hearing has brought me closer to some people	Participants did not feel this question was relevant to their situation	*That's a difficult one to answer. Not sure that's worth keeping to be honest, because it doesn't really, it's not. To be my hearing loss upon me, closer to some people*. (TA7, male, 50‐79 years)	Removed
		‘Closer’ too ambiguous	*It's your shadow in. I would say it has my hearing loss. Brought me closer to the people. I would say no, not really. (TA7, male, 50*–*79 years)*	
			*I find this is a bit of an odd question cause it's not something I've noticed. I guess you end up being a little more reliant on people. (TA6, female, 30*–*49 years)*	
Clarity	People don't seem to realise when I have not heard what they have heard	Participants found the wording confusing wording and had to read a few times	*People don't necessarily react to the fact that I'm not picking up what they're saying or I'm not hearing what they've heard. I find that one a little more difficult purely because I find it difficult to answer for someone else. (TA6, female, 30*–*49 years)*	Removed
		Participants found it difficult to answer because it was asking about someone else's perception rather than their own experience.	*That's a tricky one. I mean, people are not aware of your deafness. How could they be, and the degree of it. I mean, deafness comes in every possible degree, shade, so how can they be? (TA3, male, 80+ years)*	
Clarity	Sometimes when I'm with people I hear really well	Question was hard to understand. Participants felt that the answer would vary depending on the context/environment, situation and speaker (e.g. participants sought clarification as to whether it was a group situation or one to one)	*It's dependent on the situation, where you are, the environment and the person. I belong to a church, and some of those people in that church have got really quiet voices, and accents, and they don't open their mouth so I can't lip‐read. (TA8, female, 80+ years)*	Removed
			*I still don't really understand the question. Like is it like with some people like with my close, say with my spouse or with my mum. I didn't really get it. (TA9, male, 30*–*49 years)*	
Clarity	I understand why I don't hear the way I used to	Participants found this question ambiguous, and it was unclear what it was asking.	*I don't understand why I hear the way I do, but I think it's an ageing process. I was told that so is everything. I don't think that fits that question. I think you just accept the fact that you know you losing your hearing. (TA12, female, 50*–*79 years)*	Amended I understand why I hear the way I do (after the first wave of interviews)
		Felt that the scoring options did not work well with the question	*That's a question you can't say ‘all’, you can't answer ‘very often’, you can't answer ‘always’, ‘most of the time’. I think one must refine them, some questions you can say always, most of the time, but others you have to think what the reply will be. (TA4, female, 50*–*79 years)*	I notice the problems caused by not hearing (final survey)
Clarity	Family and friends are sensitive to my hearing needs	The term 'sensitive’ generated confusion and could be interpreted in different ways	*Family and friends are sensitive to my hearing needs. I could read that two ways cause I could either read it. Family and friends are sensitive, meaning they're being caring, or they're sensitive in that they don't like me to mention it (TA5, female, 50*–*79 years)*	Removed
			*I'm wondering if there's any easier way to ask that question. Cause if you said ‘never’, I think it's kind of mean because I'd feel a little cruel because it's not that they're not necessarily sensitive, maybe they're not fully aware of your situation. (TA6, female, 30*–*49 years)*	
Acceptability	I worry that people think I'm stupid because of my hearing	Concern that terminology might be offensive and insensitive	*Stupid is quite a loaded term. It's not the kind of word I'd expect to see on a healthcare questionnaire. (TA1, male, 16*–*29 years)*.	Amended
	I worry that people will question my intelligence because of my hearing		*If you get someone that's particularly sensitive, they might interpret this question as people think you're stupid because you can't hear. (TA6, female, 30*–*49 years)*	I worry what people think of me because I can't hear everything
Overlapping concepts	I know there are practical solutions out there to help me, such as captions or Bluetooth devices	Participants asked for clarification for sub‐titles.	*It's like not really at always or never question. I'm not sure if that question really works with the always never. (TA11, female, 16*–*29 years)*	Removed
		Did not feel the scoring worked well for this question.	*I know there are practical solutions out there to help me such as captions or Bluetooth devices, again it's either yes or no. (TA4, female, 50*–*79 years)*	
Overlapping concepts	There are times when I need help from others with my hearing	Participants found it difficult to interpret what type of ‘help’	*There are times when I need help from others with my hearing, I don't understand that question. Oh I suppose that's turning to somebody and saying, what did he say? (TA4, female, 50*–*79 years)*	Removed
		Question too broad		
Overlapping concepts	I wonder what my future life will be like with my hearing	Participants preferred a similar item asking about worry about hearing in the future	*I don't really understand that question. I've gotta be honest. As you get older, it's gonna be. It's a progressive deterioration. (TA12, female, 50*–*79 years)*	Removed
		Item had to be re‐read a few times		
		Some participants were accepting/fatalistic about hearing progression	*Do you know, I don't. Somehow, I have faith in technology and think they might find me a better hearing aid. I don't worry about it getting so bad I can't hear anything. (TA2, female, 80+ years)*	

#### 3.2.3 Relevance

Overall, most of the items seemed relevant to participants and resonated with their experiences of hearing loss. Participants were able to respond to the questions easily and relate them to their individual circumstances. However, a few items were considered irrelevant to some participants and not representative of their experiences. For example, most participants found that an item about feeling detached from the world because of missing out on news broadcasts was irrelevant to them given that radio required extra listening effort. They tended to access news via other media (e.g., social media). Some commented that they felt more ‘worried’ than ‘detached’ about missing out on important information (e.g., not hearing announcements at train stations).

Most participants viewed the emotion and identity items as relevant and important. Only a few participants seemed to consider these items less relevant as they felt their self‐confidence enabled them to cope.

I'm a rather gung‐ho sort of person so I don't let these things worry me. I'm not a little nervous old lady, not yet.

(TA4, female, 50–79 years)

Another item, ‘My hearing has brought me closer to some people’, was judged as irrelevant by some participants. When probed further, participants said they did not understand what the term ‘closer’ meant, and therefore found it hard to relate to. One participant felt that items linked to concepts of identity (‘I don't feel the way I used to about myself now I don't hear so well’) and understanding of hearing loss (‘I understand why I don't hear the way I used to’) were not relevant to their situation because they had experienced hearing loss since birth and therefore had not known anything different.

I understand why I don't hear the way I used to. Well, that I'd say it's not really relevant to me because my hearing's always stay the same.

(TA10, female, age range 16–29 years)

Based on the cognitive interviews regarding relevance, two items were removed (Table 5).

#### 3.2.4 Clarity

Most PREM items were described as clear and easy to understand. Participants were able to interpret the five graded response options consistently (always, most of the time, sometimes, rarely, never) and explain them with specific examples. Importantly, we found there was variation in participants’ responses across the items and they made use of the graded options. Participants felt some items lent themselves better to dichotomous yes/no responses. For example, items asking whether participants would like more information about devices (such as alarms, phone or TV) to support their hearing were reworded to *‘*I have thought about getting devices to help me hear alarms, the phone or TV*’*.

Participants were instructed to respond within a specific timeframe (e.g., ‘Over the last week’). However, early interviews indicated that participants needed reminders about this timeframe. Some found the timeframe helpful for answering questions (e.g., ‘Over the last week, I have felt unsafe when I am out and about because I don't hear what is going on around me’), but one participant expressed concern that it would only provide a ‘small snapshot’ of their lived experience. Ultimately, we decided to retain the 1‐week timeframe given respondents are more likely to accurately recall their experiences from the past week than from longer periods.

Some items generated confusion due to a lack of conceptual clarity and ambiguous phrasing. An item asking about other people's awareness when they had not heard something was described as ‘grammatically confusing’, and difficult to answer about someone else's perception. A few questions were found difficult to answer because they lacked context relating to the environment and/or speaker (e.g., ‘I'm always working to make sense of conversation’ and ‘I struggle in social situations because of my hearing’). Several participants commented that some situations require more effort than others (e.g., group conversations, quieter voices, noisy environments and high frequencies). We observed that participants seemed to take longer to respond to items asking about their interactions with healthcare professionals due to difficulties recalling their encounters. Some participants mentioned that they had not seen an audiologist for 2–3 years. In contrast, items more focused on the everyday experience of living with hearing loss (e.g., interacting with others and psychosocial impact) seemed to facilitate faster recall.

The challenge I would submit to this question (‘I struggle in social situations because of my hearing’) is that social situations can be very different for different people, so it could be that a social could be one to one in a quiet room for some people, or it could be in a nightclub.

(TA1, male, age range 16–29 years)

A few participants thought that the item asking how sensitive family and friends were in relation to their hearing needs could be variously interpreted. The term ‘sensitive’ could be interpreted as family and friends trying to be sensitive by not mentioning hearing loss or as kindness by acknowledging their hearing loss. The content of this item was viewed as being captured by others (e.g., ‘My family and friends support me with my hearing’), so it was removed.

The term ‘healthcare professional’ sometimes generated confusion as to whether it referred to healthcare professionals in general (e.g., GPs and nurses) or those working in audiology. Preferences for terminology varied, with some participants favouring a broader term to account for different service delivery models (such as services led by audiologists, ENT registrars, hearing aid dispensers or health scientists). Conversely, others found specific terms clearer, and some languages do not have varied terms, often referring to various healthcare professionals simply as ‘Doctors.’ To address this, we decided to use multiple terms for items used in the wider quantitative survey to examine which wording has the best response. There was also confusion about whether to base responses on experiences with or without hearing aids. Participants were instructed to reflect on their activities over the past week.

Based on the cognitive interviews regarding clarity, seven items were removed (note that three of these items were removed due to both clarity and overlapping concepts), and 12 items were amended.

#### 3.2.5 Acceptability

Overall, most participants spoke positively about My Hearing PREM and considered it appropriate and acceptable. They thought it would offer a good opportunity for patients to reflect on their situation and consider how hearing loss was impacting their lives. Most participants seemed to value items related to the emotional aspects of hearing loss (e.g., feeling left out) and how it affected their communication with others (e.g., confidence in telling others). One participant reported that although items about progressive hearing loss triggered an emotional response, they were important to include.

I think it's pretty important. I mean that's one of the biggest emotional aspects of it. I can feel my eyes welling up … certainly it's confronting … which I think is a good thing.

(TA9, male, 30–49 years)

Items that asked respondents to consider whether others questioned their intelligence due to their hearing were described as potentially offensive. The item was rephrased to ‘I worry what people think of me because I can't hear everything’. Like PPIE feedback, a few participants said that they valued a balance of positive and negative statements, although one participant felt more of the items should be framed negatively to reflect a more realistic representation of their experience. This perspective represented only one viewpoint and so a balance of negative and positive statements was retained.

It's good to have a balance, but if it is like weighted more towards negativity cause, that's pragmatic and truthful.

(TA9, male, 30–49 years)

Based on the cognitive interviews regarding acceptability, one item was removed.

#### 3.2.6 Comprehensiveness

Overall, My Hearing PREM was judged to be comprehensive by capturing core aspects of the experience. It also prompted them to reflect on aspects of their hearing loss they may not have considered otherwise.

The questions made me think about certain things that I perhaps wouldn't have just come up straight away. It gives you the sort of prompts to start a conversation

(TA10, female, age range 16–29 years)

One participant questioned why none of the items focused on hearing aids, highlighting the earlier confusion about how to respond. This emphasised the importance of explaining the concept and purpose of My Hearing PREM and how it complements existing questionnaires, such as PROMs focused on hearing aid outcomes. Another participant felt that the questionnaire lacked items addressing the experience of hearing loss in the workplace. Considering that hearing loss is more common in older generations (65+ years), we decided not to include items related to the workplace as they might not be relevant to all respondents.

To be honest it's a questionnaire about the hearing aids themselves which would probably resonate more. If I didn't use my hearing aids my answers would be totally different.

(TA4, female, 50–79 years)

Based on the interviews about comprehensiveness, two additional items were added to My Hearing PREM: one measuring perceived confidence in background noise, and another assessing whether healthcare professionals clearly explained audiology tests and results.

#### 3.2.7 Overlapping Concepts

Overall, eight items were thought to overlap conceptually with other items and were removed (three of these items were removed due to both clarity and overlapping concepts). For example, an item identifying awareness of practical solutions (e.g., subtitles and Bluetooth devices) to support hearing was considered to overlap with a similar item about the need for more information about devices. Seven items measuring worry about how hearing might progress in the future, what others think of them, and whether they had help from healthcare professionals and others were considered redundant and removed.

Following the cognitive interviews, 15 out of the 40 items were removed, and two new items were added. This left 27 items in total, with 12 of them revised for clarity. The refined set of 27 items was then tested in the subsequent phase (results to be reported separately).

#### 3.2.8 Readability Assessment

The readability analysis of the remaining 27 items demonstrated a Flesch–Kincaid reading ease of 70.4 (out of a possible 100), with a Flesch reading level of grade 6.2 (reading age 12–13 years old).

## 4 Discussion

We have described the item generation and content evaluation of My Hearing PREM, the first measure internationally to assess the experience of living with hearing loss and audiology care. The preliminary set of items was informed by a literature synthesis [51] and our conceptual model [45]. Interviews with adults living with hearing loss, together with ongoing input from the PPIE groups and key stakeholders, contributed to the revision and refinement of items. My Hearing PREM will be further validated and refined in a quantitative study using conventional and modern (Rasch) psychometric analysis techniques [64].

The results indicate that most of the PREM content was relevant, clear, acceptable and comprehensive. The multiple iterations and versions denote the value of deploying a robust approach to developing and testing the novel measure. Interviews uncovered elements (e.g., irrelevant questions, ambiguous wording) that may reduce the likelihood of respondents completing it and affect the quality of data collected [38, 47]. Our research contributes to a small, albeit growing body of scale development in hearing which draws on international standards (i.e., COSMIN criteria) [38, 48, 65, 66]. To date, only hearing‐specific PROMs [38, 48] have been developed, and there is a lack of guidance to support the development of high‐quality, psychometrically sound PREMs. Therefore, our work reinforces the need for PREM‐specific guidance [40, 67].

The content evaluation process highlighted several problems that participants encountered when answering items that informed item rewording or removal. Initially, items were worded negatively based on our qualitative research which highlighted the ongoing struggles experienced [45, 52]. However, some participants and PPIE respondents felt the PREM should include a mixture of positive and negative statements. There is conflicting evidence over whether including reverse‐worded items (i.e., items that are phrased in the opposite direction to other items) is important. Some studies have found that reverse‐worded items reduce response bias, including acquiescence (the tendency for respondents to agree with items, without the action being a true reflection of their own views) and inattention (responding to items inconsistently without paying attention to item content) [68, 69]. Other hearing research has found that reverse‐worded items can generate confusion and careless answering [48, 70]; however, we note that in one study participants had to switch between positively and negatively worded items, which used different response scales [48]. Based on participant feedback and the fact we were using one single response scale, we used a combination of positively and negatively worded questions.

In line with previous work, we found that items tapping into the emotional burden of hearing loss appeared to resonate more with some participants compared with others [48]. Evidence consistently shows that hearing loss adversely impacts well‐being [10, 71]. The resources (e.g., psychological, social support and health literacy) that people draw upon to buffer the emotional impact can help to explain differences in experiences. Those with more resources may have greater capacity to manage their hearing loss [52]. This supports our decision to take a lifeworld‐led, holistic approach to understanding hearing loss and the focus we have taken with My Hearing PREM on subjective experience by drawing out expressions of emotional burden, communication challenges and social support.

Our cognitive interviews indicate that it is important to be mindful of My Hearing PREM triggering emotions. Participants appreciated questions that addressed emotions but noted they evoked challenging feelings, such as worry and uncertainty. This echoes findings from cognitive interviews evaluating the Social Participation Restrictions Questionnaire, where participants described the measure as thought‐provoking and suggested that questionnaires should include details on support services [48].

Although participants were recruited from regions with diverse areas of affluence and poverty, information about education, occupation, type of hearing loss or existing chronic conditions was not collected. These characteristics are likely to shape participants’ responses and their ability to complete the PREM. The ratings of relevance, clarity, acceptability and comprehensiveness for the PREM items may not be representative of all patient perspectives. Most participants were interviewed twice by the same researcher. Although participants were asked to express their honest opinions, it is possible that some participants felt uncomfortable providing critical feedback to a researcher with whom they had built a rapport and had been involved with developing the PREM [72]. We also acknowledge that due to time limitations, only one researcher was able to analyse the data. Ideally, multiple researchers should have been involved in analysing the data to enhance rigour. However, we did hold regular meetings to discuss the data and observation notes.

My Hearing PREM is designed to be completed by adults (aged 16 years and over) at any stage of their hearing health journey. We envisage My Hearing PREM to be used to monitor patient experience over time, from one appointment to another, to see how responsive the measure is in detecting clinically relevant changes in experience (e.g., emotions and confidence in social interactions). It could be used to identify whether a person's experience changes if they attend hearing therapy or have a hearing aid fitted. Like other studies, we did not ask participants to comment on the potential responsiveness of the PREM (i.e., capable of detecting clinically meaningful changes in the lived experience of hearing loss) [38, 48]. Our future quantitative work will be able to assess the responsiveness of My Hearing PREM. Ultimately, My Hearing PREM will be free to use and accessible to all hearing care professionals. It will be adopted onto the existing patient management system software to facilitate routine clinical access.

## 5 Conclusion

There is currently no hearing loss‐specific PREM in audiology. This article describes the different phases involved in developing and evaluating the content of a novel PREM focused on the lived experience of hearing loss and audiology care. This work underlines the importance of using a rigorous and iterative approach to PREM development to identify any potential problems with the measure and improve its relevance, clarity, comprehensiveness and acceptability. PREM development guidelines are required to promote PREMs across healthcare; our experience means we are well placed to create them. The next phase of this research is to conduct a quantitative survey to further evaluate the psychometric properties of the PREM against existing measures and reduce the items using modern psychometric Rasch analyses. Our vision for the PREM is to facilitate dialogue between patients and staff about the patient experience and illuminate gaps in audiology service provision so that we might achieve the goal of integrating both patient‐centred and lifeworld‐led approaches into audiology care.

## Author Contributions

**Sian K. Smith:** conceptualisation, methodology, investigation, validation, formal analysis, visualisation, project administration, writing–original draft, writing–review and editing. **Georgina Burns O'Connell:** conceptualisation, methodology, investigation, validation, formal analysis, visualisation, project administration, writing–review and editing. **Saira Hussain:** methodology, investigation, validation, formal analysis, project administration, writing–review and editing. **Rebecca Knibb:** conceptualisation, methodology, funding acquisition, visualisation, writing–review and editing. **Rosemary Greenwood:** conceptualisation, methodology, funding acquisition, writing–review and editing. **Rachel Shaw:** conceptualisation, methodology, funding acquisition, writing–review and editing. **Jean Straus:** conceptualisation, methodology, investigation, formal analysis, resources, visualisation, writing–review and editing. **Jonathan Banks:** conceptualisation, methodology, funding acquisition, writing–review and editing. **Amanda Hall:** conceptualisation, methodology, funding acquisition, visualisation, writing–review and editing. **Nisha Dhanda:** conceptualisation, methodology, investigation, writing–review and editing. **Sian Noble:** conceptualisation, methodology, funding acquisition, writing–review and editing. **Helen Pryce:** conceptualisation, methodology, investigation, validation, supervision, formal analysis, resources, funding acquisition, visualisation, project administration, writing–review and editing.

## Ethics Statement

The study was approved by the West of Scotland Research Ethics Service (approval date: 6 May 2022; ref22/WS/0057) and the Health Research Authority and Health and Care Research Wales (HCRW) (approval date: 14 June 2022; IRAS project ID: 308816).

## Conflicts of Interest

The authors declare no conflicts of interest.

## Supporting information

S1. Cognitive interview schedule.

## Data Availability

Research data are not shared.

## References

[1] World Health Organization ., World Report on Hearing (Geneva, Switzerland: World Health Organization, 2021), https://www.who.int/publications/i/item/9789240020481.

[2] B. S. Wilson and D. L. Tucci , “Addressing the Global Burden of Hearing Loss,” Lancet 397, no. 10278 (2021): 945–947, 10.1016/S0140-6736(21)00522-5. 33714376

[3] J. A. Holman , A. Drummond , S. E. Hughes , and G. Naylor , “Hearing Impairment and Daily‐Life Fatigue: A Qualitative Study,” International Journal of Audiology 58, no. 7 (2019): 408–416, 10.1080/14992027.2019.1597284. 31032678 PMC6567543

[4] C. R. May , D. T. Eton , K. Boehmer , et al., “Rethinking the Patient: Using Burden of Treatment Theory to Understand the Changing Dynamics of Illness,” BMC Health Services Research 14, no. 1 (2014): 281, 10.1186/1472-6963-14-281. 24969758 PMC4080515

[5] H. Pryce , A. Hall , A. Laplante‐Lévesque , and E. Clark , “A Qualitative Investigation of Decision Making during Help‐Seeking for Adult Hearing Loss,” International Journal of Audiology 55, no. 11 (2016): 658–665, 10.1080/14992027.2016.1202455. 27385528

[6] A. Pike , S. Moodie , K. Parsons , et al., “Something Is Just Not Right With My Hearing”: Early Experiences of Adults Living With Hearing Loss,” International Journal of Audiology 61, no.9 (2022): 787–797, 10.1080/14992027.2021.1983656. 34612131

[7] E. Heffernan , C. M. Withanachchi , and M. A. Ferguson , “The Worse My Hearing Got, the Less Sociable I Got’: A Qualitative Study of Patient and Professional Views of the Management of Social Isolation and Hearing Loss,” Age and Ageing 51, no. 2 (2022), 10.1093/ageing/afac019.

[8] A. Shukla , M. Harper , E. Pedersen , et al., “Hearing Loss, Loneliness, and Social Isolation: A Systematic Review,” Otolaryngology–Head and Neck Surgery 162, no. 5 (2020): 622–633, 10.1177/0194599820910377. 32151193 PMC8292986

[9] R. J. Bennett , L. Saulsman , R. H. Eikelboom , and M. Olaithe , “Coping With the Social Challenges and Emotional Distress Associated With Hearing Loss: A Qualitative Investigation Using Leventhal's Self‐Regulation Theory,” International Journal of Audiology 61, no. 5 (2022): 353–364, 10.1080/14992027.2021.1933620. 34148485

[10] J. A. Holman , Y. H. K. Ali , and G. Naylor , “A Qualitative Investigation of the Hearing and Hearing‐Aid Related Emotional States Experienced by Adults With Hearing Loss,” International Journal of Audiology 62, no. 10 (2022): 973–982, 10.1080/14992027.2022.2111373. 36036164

[11] H. Pryce and A. Hall , “The Role of Shared Decision‐Making in Audiologic Rehabilitation,” Perspectives on Aural Rehabilitation and Its Instrumentation 21, no. 1 (2014): 15–23.

[12] B. J. Parmar , K. Mehta , D. A. Vickers , and J. K. Bizley , “Experienced Hearing Aid Users’ Perspectives of Assessment and Communication Within Audiology: A Qualitative Study Using Digital Methods,” International Journal of Audiology 61, no. 11 (2022): 956–964, 10.1080/14992027.2021.1998839. 34821527 PMC7614704

[13] S. T. Skou , F. S. Mair , M. Fortin , et al., “Multimorbidity,” Nature Reviews Disease Primers 8, no. 1 (2022): 48, 10.1038/s41572-022-00376-4. PMC7613517 35835758

[14] K. R. Boehmer , M. R. Gionfriddo , R. Rodriguez‐Gutierrez , et al., “Patient Capacity and Constraints in the Experience of Chronic Disease: A Qualitative Systematic Review and Thematic Synthesis,” BMC Family Practice 17, no. 1 (2016): 127, 10.1186/s12875-016-0525-9. 27585439 PMC5009523

[15] “Hearing Loss in Adults: Assessment and Management NICE Guideline (NG98),” National Institute of Clinical Excellence (NICE), 2018, www.nice.org.uk/guidance/ng98. 30011159

[16] “Practice Guidance Common Principles of Rehabilitation for Adults in Audiology Services Date,” British Society of Audiology, 2016, https://wwwthebsa.org.uk/wp-content/uploads/2023/10/OD104-52-Practice-Guidance-Common-Principles-of-Rehabilitation-for-Adults-in-Audiology-Services-.pdf.

[17] C. Grenness , L. Hickson , A. Laplante‐Lévesque , and B. Davidson , “Patient‐Centred Care: A Review for Rehabilitative Audiologists,” International Journal of Audiology 53, no. Suppl 1 (2014): S60–S67, 10.3109/14992027.2013.847286. 24447236

[18] C. Coleman , K. Muñoz , C. Ong , G. Butcher , L. Nelson , and M. Twohig , “Opportunities for Audiologists to Use Patient‐Centered Communication During Hearing Device Monitoring Encounters,” Seminars in Hearing 39, no. 1 (2018): 032–043, 10.1055/s-0037-1613703. PMC5802989 29422711

[19] Department of Health and Social Care , NHS England, *Liberating the NHS: No decision About me, Without Me* . (London: NHS, 2012).

[20] “Hearing Loss in Adults: Assessment and Management NICE guideline (NG98),” National Institute of Clinical Excellence (NICE), 2018, www.nice.org.uk/guidance/ng98. 30011159

[21] A. C. Coulter , “Making Shared Decision‐Making A Reality: No Decision About Me, Without Me,” *The King's Fund*, 2011.

[22] IDA Institute . “Person‐Centered Care Definitions,” IDA Institute (2019), https://idainstitute.com/what_we_do/pcc_definitions.

[23] J. S. King , M. H. Eckman , and B. W. Moulton , “The Potential of Shared Decision Making to Reduce Health Disparities,” Journal of Law, Medicine & Ethics 39, no. Suppl 1 (2011): 30–33, 10.1111/j.1748-720X.2011.00561.x. 21309892

[24] D. Stacey , F. Légaré , K. Lewis , et al., “Decision Aids for People Facing Health Treatment or Screening Decisions,” Cochrane Database of Systematic Reviews 4, no. 4 (2017): 001431, 10.1002/14651858.CD001431.pub5. PMC6478132 28402085

[25] L. T. Walseth and E. Schei , “Effecting Change Through Dialogue: Habermas’ Theory of Communicative Action as a Tool in Medical Lifestyle Interventions,” Medicine, Health Care and Philosophy 14, no. 1 (2011): 81–90, 10.1007/s11019-010-9260-5. 20552281

[26] C. Ellis‐Hill , C. Pound , and K. Galvin , “Making the Invisible More Visible: Reflections on Practice‐Based Humanising Lifeworld‐Led Research – Existential Opportunities for Supporting Dignity, Compassion and Wellbeing,” Scandinavian Journal of Caring Sciences 36, no. 4 (2022): 1037–1045, 10.1111/scs.13013. 34169563

[27] K. Dahlberg , L. Todres , and K. Galvin , “Lifeworld‐Led Healthcare is More Than Patient‐Led Care: An Existential View of Well‐Being,” Medicine, Health Care and Philosophy 12, no. 3 (2009): 265–271, 10.1007/s11019-008-9174-7. 19101822

[28] J. Zhang , “Fostering Dialogue: A Phenomenological Approach to Bridging the Gap Between the ‘Voice of Medicine’ and the ‘Voice of the Lifeworld,’” Medicine, Health Care and Philosophy 27, no. 2 (2024): 155–164, 10.1007/s11019-024-10195-x. 38285166

[29] H. Pryce and R. Shaw , “Lifeworld Interpretation of Tinnitus,” Medical Humanities 45, no. 4 (2019): 428–433, 10.1136/medhum-2019-011665. 31235652

[30] H. Pryce , S. K. Smith , G. Burns‐O'Connell , et al., “Protocol for the Development and Validation of a Patient‐Reported Experience Measure (PREM) for People With Hearing Loss: The PREM‐HeLP,” BMJ Open 13, no. 11 (2023): 075229, 10.1136/bmjopen-2023-075229. PMC10689349 38030247

[31] H. Pryce , S. K. Smith , G. Burns‐O'connell , et al., “Protocol for a Qualitative Study Exploring the Lived Experience of Hearing Loss and Patient Reported Experience in the UK: The HeLP Study,” BMJ Open 13, no. 6 (2023): 069363, 10.1136/bmjopen-2022-069363. PMC10254947 37286313

[32] M. Hodson , S. Andrew , and C. Michael Roberts , “Towards an Understanding of PREMS and PROMS in COPD,” Breathe 9 (2013): 358–364, 10.1183/20734735.006813.

[33] A. M. Stover , L. Haverman , H. A. van Oers , et al., “Using an Implementation Science Approach to Implement and Evaluate Patient‐Reported Outcome Measures (PROM) Initiatives in Routine Care Settings,” Quality of Life Research 30, no. 11 (2021): 3015–3033, 10.1007/s11136-020-02564-9. 32651805 PMC8528754

[34] R. Windle , “Trends in COSI Responses Associated With Age and Degree of Hearing Loss,” International Journal of Audiology 61, no. 5 (2022): 416–427, 10.1080/14992027.2021.1937347. 34137647

[35] K. Viergever , J. T. Kraak , E. M. Bruinewoud , J. C. F. Ket , S. E. Kramer , and P. Merkus , “Questionnaires in Otology: A Systematic Mapping Review,” Systematic Reviews 10, no. 1 (2021): 119, 10.1186/s13643-021-01659-9. 33879248 PMC8059288

[36] S. E. Hughes , F. L. Rapport , I. Boisvert , C. M. McMahon , and H. A. Hutchings , “Patient‐Reported Outcome Measures (Proms) for Assessing Perceived Listening Effort in Hearing Loss: Protocol for a Systematic Review,” BMJ Open 7, no. 5 (2017): e014995, 10.1136/bmjopen-2016-014995. PMC5734199 28592576

[37] E. Heffernan , D. W. Maidment , J. G. Barry , and M. A. Ferguson , “Refinement and Validation of the Social Participation Restrictions Questionnaire: An Application of Rasch Analysis and Traditional Psychometric Analysis Techniques,” Ear and Hearing 40, no. 2 (2019): 328–339. 29905669 10.1097/AUD.0000000000000618 PMC7617168

[38] S. Gotowiec , R. J. Bennett , J. Larsson , and M. Ferguson , “Development of a Self‐Report Measure of Empowerment Along the Hearing Health Journey: A Content Evaluation Study,” International Journal of Audiology 63 4 (2023): 275–285. 36794384 10.1080/14992027.2023.2174456

[39] R. J. Bennett , J. Larsson , S. Gotowiec , and M. Ferguson , “Refinement and Validation of the Empowerment Audiology Questionnaire: Rasch Analysis and Traditional Psychometric Evaluation,” Ear and Hearing 45, no. 3 (2024): 583–599, 10.1097/aud.0000000000001449. 38082487 PMC11008442

[40] C. Bull , J. Byrnes , R. Hettiarachchi , and M. Downes , “A Systematic Review of the Validity and Reliability of Patient‐Reported Experience Measures,” Health Services Research 54, no. 5 (2019): 1023–1035, 10.1111/1475-6773.13187. 31218671 PMC6736915

[41] B. Graham , J. E. Smith , F. Barham , and J. M. Latour , “Involving Patients and Caregivers to Develop Items for a New Patient‐Reported Experience Measure for Older Adults Attending the Emergency Department. Findings From a Nominal Group Technique Study,” Health Expectations: An International Journal of Public Participation in Health Care and Health Policy 26, no. 5 (2023): 2040–2049, 10.1111/hex.13811. 37391897 PMC10485325

[42] E. M. Sheldon , G. Lillington , K. Simpson , et al., “Development of an Inflammatory Bowel Disease (IBD) Patient‐Reported Experience Measure (PREM): A Patient‐Led Consensus Work and ‘Think Aloud’ Study for a Quality Improvement Programme,” Health Expectations 26, no. 1 (2023): 213–225, 10.1111/hex.13647. 36335578 PMC9854292

[43] C. Bull , H. Teede , and D. Watson , “Selecting and Implementing Patient‐Reported Outcome and Experience Measures to Assess Health System Performance,” JAMA Health Forum 3 4 (2022): e220326. 36218960 10.1001/jamahealthforum.2022.0326

[44] K. Withers , R. Palmer , S. Lewis , and G. Carolan‐Rees , “First Steps in PROMs and PREMs Collection in Wales as Part of the Prudent and Value‐Based Healthcare Agenda,” Quality of Life Research: An International Journal of Quality of Life Aspects of Treatment, Care and Rehabilitation 30 (2021): 3157–3170. 33249539 10.1007/s11136-020-02711-2 PMC7700742

[45] H. Pryce , S. Smith , G. Burns O'Connell , S. Hussain , J. Straus , and R. Shaw , “The Lived Experience of Hearing Loss—An Individualised Responsibility,” International Journal of Audiology (2024): 1–9, 10.1080/14992027.2024.2351037. 38767328

[46] L. B. Mokkink , C. B. Terwee , D. L. Patrick , et al., “The COSMIN Checklist for Assessing the Methodological Quality of Studies on Measurement Properties of Health Status Measurement Instruments: An International Delphi Study,” Quality of Life Research 19, no. 4 (2010): 539–549, 10.1007/s11136-010-9606-8. 20169472 PMC2852520

[47] M. Brod , L. E. Tesler , and T. L. Christensen , “Qualitative Research and Content Validity: Developing Best Practices Based on Science and Experience,” Quality of Life Research 18, no. 9 (2009): 1263–1278, 10.1007/s11136-009-9540-9. 19784865

[48] E. Heffernan , N. S. Coulson , and M. A. Ferguson , “Development of the Social Participation Restrictions Questionnaire (SPaRQ) Through Consultation With Adults With Hearing Loss, Researchers, and Clinicians: A Content Evaluation Study,” International Journal of Audiology 57, no. 10 (2018): 791–799, 10.1080/14992027.2018.1483585. 29966457

[49] B. B. Reeve , K. W. Wyrwich , A. W. Wu , et al., “ISOQOL Recommends Minimum Standards for Patient‐Reported Outcome Measures Used in Patient‐Centered Outcomes and Comparative Effectiveness Research,” Quality of Life Research 22 (2013): 1889–1905, 10.1007/s11136-012-0344-y. 23288613

[50] S. L. Belvedere and N. A. de Morton , “Application of Rasch Analysis in Health Care Is Increasing and Is Applied for Variable Reasons in Mobility Instruments,” Journal of Clinical Epidemiology 63, no. 12 (2010): 1287–1297, 10.1016/j.jclinepi.2010.02.012. 20971422

[51] H. Pryce , S. K. Smith , G. Burns‐O'Connell , and R Shaw , “The Lived Experience of Hearing Loss: A Systematic Review With Narrative Synthesis,” (poster presentation, BSA Scientific Meeting, Birmingham, UK, 2024). 10.1080/14992027.2025.2523902 40588153

[52] S. K. Smith , H. Pryce , G. B. O'Connell , S. Hussain , R. Shaw , and J. Straus , “‘The Burden Is Very Much on Yourself’: A Qualitative Study to Understand the Illness and Treatment Burden of Hearing Loss Across the Life Course,” Health Expectations 27, no. 3 (2024): e14067, 10.1111/hex.14067. 38715316 PMC11076985

[53] R. Flesch , “A New Readability Yardstick,” Journal of Applied Psychology 32, no. 3 (1948): 221–233. 18867058 10.1037/h0057532

[54] J. P. Kincaid , R. P. Fishburne Jr. , R. L. Rogers , and B. S. Chissom , “Derivation of New Readability Formulas (Automated Readability Index, Fog Count and Flesch Reading Ease Formula) for Navy Enlisted Personnel,” Institute for Simulation and Training, 56, (1975), https://stars.library.ucf.edu/istlibrary/56.

[55] K. Bould and M. J. Forshaw , “Readability of Online COVID‐19 Health Information and Advice,” International Journal of Health Promotion and Education 61, no. 4 (2023): 189–209, 10.1080/14635240.2022.2098160.

[56] I. Oosthuizen , L. M. S. Kumar , K. V. Nisha , et al., “Patient‐Reported Outcome Measures for Hearing Aid Benefit and Satisfaction: Content Validity and Readability,” Journal of Speech, Language, and Hearing Research 66, no. 10 (2023): 4117–4136, 10.1044/2023_jslhr-22-00535. 37708535

[57] C. Priede and S. J. I. Jo. S. R. M. Farrall , “Comparing Results From Different Styles of Cognitive Interviewing:‘Verbal ProBing'vs.‘Thinking Aloud,” International Journal of Social Research Methodology 14 4 (2011): 271–287.

[58] C. Buers , M. Triemstra , E. Bloemendal , N. C. Zwijnenberg , M. Hendriks , and D. M. J. Delnoij , “The Value of Cognitive Interviewing for Optimizing a Patient Experience Survey,” International Journal of Social Research Methodology 17, no. 4 (2014): 325–340, 10.1080/13645579.2012.750830.

[59] T. Boren and J. J. I Ramey , “Thinking Aloud: Reconciling Theory and Practice,” IEEE Transactions on Professional Communication 43 3 (2000): 261–278.

[60] V. Braun and V. J. Qrip Clarke , “Using Thematic Analysis in Psychology,” Qualitative Research in Psychology 3 2 (2006): 77–101.

[61] G. E. Marcus , W. R. Neuman , and M. B. MacKuen , “Measuring Emotional Response: Comparing Alternative Approaches to Measurement,” Political Science Research and Methods 5, no. 4 (2017): 733–754, 10.1017/psrm.2015.65.

[62] J. Straus , “What the Beeps’ Going On?” RNID Magazine, 2023: 44–45.

[63] V. K. C. Manchaiah and D. Stephens , “Perspectives on Defining ‘Hearing Loss’ and Its Consequences,” Hearing, Balance and Communication 11, no. 1 (2013): 6–16, 10.3109/21695717.2012.756624.

[64] J. J. Gagnier , J. Lai , L. B. Mokkink , and C. B. Terwee , “COSMIN Reporting Guideline for Studies on Measurement Properties of Patient‐Reported Outcome Measures,” Quality of Life Research 30, no. 8 (2021): 2197–2218, 10.1007/s11136-021-02822-4. 33818733

[65] R. J. Bennett , D. S. Taljaard , C. G. Brennan‐Jones , S. Tegg‐Quinn , and R. H. Eikelboom , “Evaluating Hearing Aid Handling Skills: A Systematic and Descriptive Review,” International Journal of Audiology 54, no. 11 (2015): 765–776, 10.3109/14992027.2015.1052104. 26076941

[66] S. L. Smith , M. Kathleen Pichora‐Fuller , K. L. Watts , and C. La More , “Development of the Listening Self‐Efficacy Questionnaire (LSEQ),” International Journal of Audiology 50, no. 6 (2011): 417–425, 10.3109/14992027.2011.553205. 21470067

[67] C. Bull , H. Teede , D. Watson , and E. J. Callander , “Selecting and Implementing Patient‐Reported Outcome and Experience Measures to Assess Health System Performance,” JAMA Health Forum 3, no. 4 (2022): e220326‐e, 10.1001/jamahealthforum.2022.0326. 36218960

[68] J. Rattray and M. C. Jones , “Essential Elements of Questionnaire Design and Development,” Journal of Clinical Nursing 16, no. 2 (2007): 234–243, 10.1111/j.1365-2702.2006.01573.x. 17239058

[69] E. Sonderen , R. Sanderman , and J. C. Coyne , “Ineffectiveness of Reverse Wording of Questionnaire Items: Let's Learn from Cows in the Rain,” PLOS ONE 8, no. 7 (2013): e68967, 10.1371/journal.pone.0068967. 23935915 PMC3729568

[70] E. B. Carlson , S. R. Smith , P. A. Palmieri , et al., “Development and Validation of a Brief Self‐Report Measure of Trauma Exposure: the Trauma History Screen,” Psychological Assessment 23, no. 2 (2011): 463–477, 10.1037/a0022294. 21517189 PMC3115408

[71] E. Heffernan , N. S. Coulson , H. Henshaw , J. G. Barry , and M. A. Ferguson , “Understanding the Psychosocial Experiences of Adults with Mild‐Moderate Hearing Loss: An Application of Leventhal's Self‐Regulatory Model,” International Journal of Audiology 55, no. Suppl 3 (2016): S3–S12, 10.3109/14992027.2015.1117663. PMC5706634 26754550

[72] M. Horsfall , M. Eikelenboom , S. Draisma , and J. H. Smit , “The Effect of Rapport on Data Quality in Face‐To‐Face Interviews: Beneficial or Detrimental?” International Journal of Environmental Research and Public Health 18, no. 20 (2021): 10858, 10.3390/ijerph182010858. 34682600 PMC8535677

