# Supplementary material for: Development of the First Patient‐Reported Experience Measure (PREM) for Hearing Loss in Audiology Care—My Hearing PREM
Source: Health Expect. 2024 Nov 6;27(6):e70088. doi: 10.1111/hex.70088 (PMC11540936; doi:10.1111/hex.70088)
Supplement: Supplementary file 1 — S1. Cognitive interview schedule. [file HEX-27-e70088-s001.docx]

**Cognitive ‘think aloud’ interview schedule**

**Aim**

To encourage participants to talk through the process of completing the PREM questionnaire and identify what worked well/ not well and areas of improvement

**Introduction**

- Introduce self and role in the study
- Reiterate aims of the study (see below)
- Confidentiality and anonymity
- Obtain consent to record

***Aims:*** We have created a questionnaire to find out more about what it is like to live with hearing loss. Other questionnaires ask about what sounds you can or cannot hear, but our questionnaire asks you about how not hearing well affects your life.

We are interested to know if the questions are easy to understand, make sense, and if we are asking the right questions about living with hearing loss. Please let us know if you have suggestions for other questions we might ask.

**Think aloud interview**

- I am sharing our draft PREM questionnaire. I would like you to fill this out but to talk me through how you interpret the items and what answer you think you’d give
- If you could answer each question in terms of thinking about how it has been over the last week (note: keep the participant on track)
- *Prompt:* You’d mark that as sometimes – can you tell me more about why you’re scoring it like that?
- *Prompt;* what does this item mean to you? How relevant is this item to you? How important is this item? How would you respond to this item/ Why?
- *Prompt:* Some participants have found this question difficult, how do you find it?

***Note:*** if you think it might be too overwhelming for participants, state that you will focus on a few items

**Make notes on:**

- Uncertainties in question and responding to questions– any questions seeking clarification, appropriate language/ phrasing etc.
- Is the scoring clear to the participant?
- Ideas for rephrasing questions
- Change in mood – comfort/ discomfort

**Questions towards the end of the ‘think aloud’ phase**

- Overall, what do you think of the questionnaire (prompt: relevance, acceptability, appropriateness, content, length, structure)?
- Is it missing anything important in your experience?
- Suggestions on how we could improve it

**After each interview**

- Review recording and note:
- Any items confuse?
- Scale confuse?
- Any items with multiple potential responses?
- Is their process straightforward or not?
- Reflexively note your influence

**After each interview – debrief**

- Send debrief email to team
- Provide team with information about participant’s characteristics
- Items that participants thought worked well/ not well/ difficulties – as recorded above
- Provide details of other interview dates.
